# Dipeptidyl-peptidase 3 and IL-6: potential biomarkers for diagnostics in COVID-19 and association with pulmonary infiltrates

**DOI:** 10.1007/s10238-023-01193-z

**Published:** 2023-09-21

**Authors:** Stephan T. Staudner, Simon B. Leininger, Manuel J. Vogel, Julian Mustroph, Ute Hubauer, Christine Meindl, Stefan Wallner, Petra Lehn, Ralph Burkhardt, Frank Hanses, Markus Zimmermann, Gregor Scharf, Okka W. Hamer, Lars S. Maier, Julian Hupf, Carsten G. Jungbauer

**Affiliations:** 1 Department of Internal Medicine II, University Hospital Regensburg, Regensburg, Germany; 2 Department of Clinical Chemistry and Laboratory Medicine, University Hospital Regensburg, Regensburg, Germany; 3 Emergency Department, University Hospital Regensburg, Regensburg, Germany; 4 Department of Infection Prevention and Infectious Diseases, University Hospital Regensburg, Regensburg, Germany; 5 Department of Radiology, University Hospital Regensburg, Regensburg, Germany

**Keywords:** COVID-19, SARS-CoV-2, Dipeptidyl-peptidase 3, IL-6, Pulmonary infiltrates, Artificial intelligence

## Abstract

Coronavirus SARS-CoV-2 spread worldwide, causing a respiratory disease known as COVID-19. The aim of the present study was to examine whether Dipeptidyl-peptidase 3 (DPP3) and the inflammatory biomarkers IL-6, CRP, and leucocytes are associated with COVID-19 and able to predict the severity of pulmonary infiltrates in COVID-19 patients versus non-COVID-19 patients. 114 COVID-19 patients and 35 patients with respiratory infections other than SARS-CoV-2 were included in our prospective observational study. Blood samples were collected at presentation to the emergency department. 102 COVID-19 patients and 28 non-COVID-19 patients received CT imaging (19 outpatients did not receive CT imaging). If CT imaging was available, artificial intelligence software (CT Pneumonia Analysis) was used to quantify pulmonary infiltrates. According to the median of infiltrate (14.45%), patients who obtained quantitative CT analysis were divided into two groups (> median: 55 COVID-19 and nine non-COVID-19, ≤ median: 47 COVID-19 and 19 non-COVID-19). DPP3 was significantly elevated in COVID-19 patients (median 20.85 ng/ml, 95% CI 18.34–24.40 ng/ml), as opposed to those without SARS-CoV-2 (median 13.80 ng/ml, 95% CI 11.30–17.65 ng/ml; *p* < 0.001, AUC = 0.72), opposite to IL-6, CRP (each *p* = n.s.) and leucocytes (*p* < 0.05, but lower levels in COVID-19 patients). Regarding binary logistic regression analysis, higher DPP3 concentrations (OR = 1.12, *p* < 0.001) and lower leucocytes counts (OR = 0.76, *p* < 0.001) were identified as significant and independent predictors of SARS-CoV-2 infection, as opposed to IL-6 and CRP (each *p* = n.s.). IL-6 was significantly increased in patients with infiltrate above the median compared to infiltrate below the median both in COVID-19 (*p* < 0.001, AUC = 0.78) and in non-COVID-19 (*p* < 0.05, AUC = 0.81). CRP, DPP3, and leucocytes were increased in COVID-19 patients with infiltrate above median (each *p* < 0.05, AUC: CRP 0.82, DPP3 0.70, leucocytes 0.67) compared to infiltrate below median, opposite to non-COVID-19 (each *p* = n.s.). Regarding multiple linear regression analysis in COVID-19, CRP, IL-6, and leucocytes (each *p* < 0.05) were associated with the degree of pulmonary infiltrates, as opposed to DPP3 (*p* = n.s.). DPP3 showed the potential to be a COVID-19-specific biomarker. IL-6 might serve as a prognostic marker to assess the extent of pulmonary infiltrates in respiratory patients.

## Introduction

The latest increase in SARS-CoV-2 cases and related death numbers in China demonstrate that the COVID-19 pandemic is far from over [1]. Efficient and appropriate allocation of limited resources in the health care system is the main task in times of rising cases. Therefore, biomarkers need to be found that detect COVID-19 patients, identify those at high-risk, and help understand the underlying pathophysiology. Eventually, this could lead to new pharmacological treatment options.

Dipeptidyl-peptidase 3 (DPP3) has a complex role in physiological metabolism involving signal transduction, pain modulation, blood pressure regulation, and immunomodulation [2, 3]. Previous literature has described it as a predictive biomarker in sepsis and severely-ill patients [4–7]. Due to the cleavage of angiotensin II, DPP3 is closely related to the renin–angiotensin–aldosterone system (RAAS), which also includes angiotensin-converting enzyme 2 (ACE2) [3, 8, 9]. The latter serves as a functional receptor for SARS-CoV-2 to enter the host cell [10, 11]. However, the existing data on the role of DPP3 in COVID-19 is restricted to critically ill patients [12, 13]. In particular, no research exists about whether DPP3 can predict COVID-19 infection.

IL-6 is a biomarker in sepsis, respiratory infection, and COVID-19 [14–19]. Previous studies have analysed the predictive value of the degree of pulmonary infiltrates in COVID-19 to clinical outcomes [20–25]. One retrospective study described IL-6 as an independent predictor for COVID-19 lung injury, measured semi-quantitatively [20]. However, there are no studies on the predictive value of IL-6 for the extent of lung infiltrates when the latter are measured quantitatively by artificial intelligence software. Additionally, the current study has used a broad emergency department collective including COVID-19 positive and COVID-19 negative respiratory patients.

Identifying molecular mechanisms and key processes in the pathophysiology of COVID-19 is crucial to find prognostic markers and drug targets. Karami et al. detected hub genes to identify pivotal pathways in SARS-CoV-2 infection [26]. The current study contributes to this search for key modules in COVID-19.

Hence, this prospective observational study examined whether DPP3 could predict the occurrence of a COVID-19 infection. Additionally, DPP3 and the inflammatory biomarkers IL-6, CRP, and leucocytes were evaluated regarding their association with the degree of pulmonary infiltrates.

## Methods

### Study population

Patients showing signs of a respiratory infection at the University Hospital Regensburg emergency department were included in this prospective observational study between March 2020 and June 2021. In addition, eligible patients were at least 18 years old and gave informed consent. The study was conducted by the guidelines of Good Clinical Practice and the standards for experiments on humans set out in the Declaration of Helsinki. The Ethics Committee of the University of Regensburg approved the study.

**Fig. 1:**
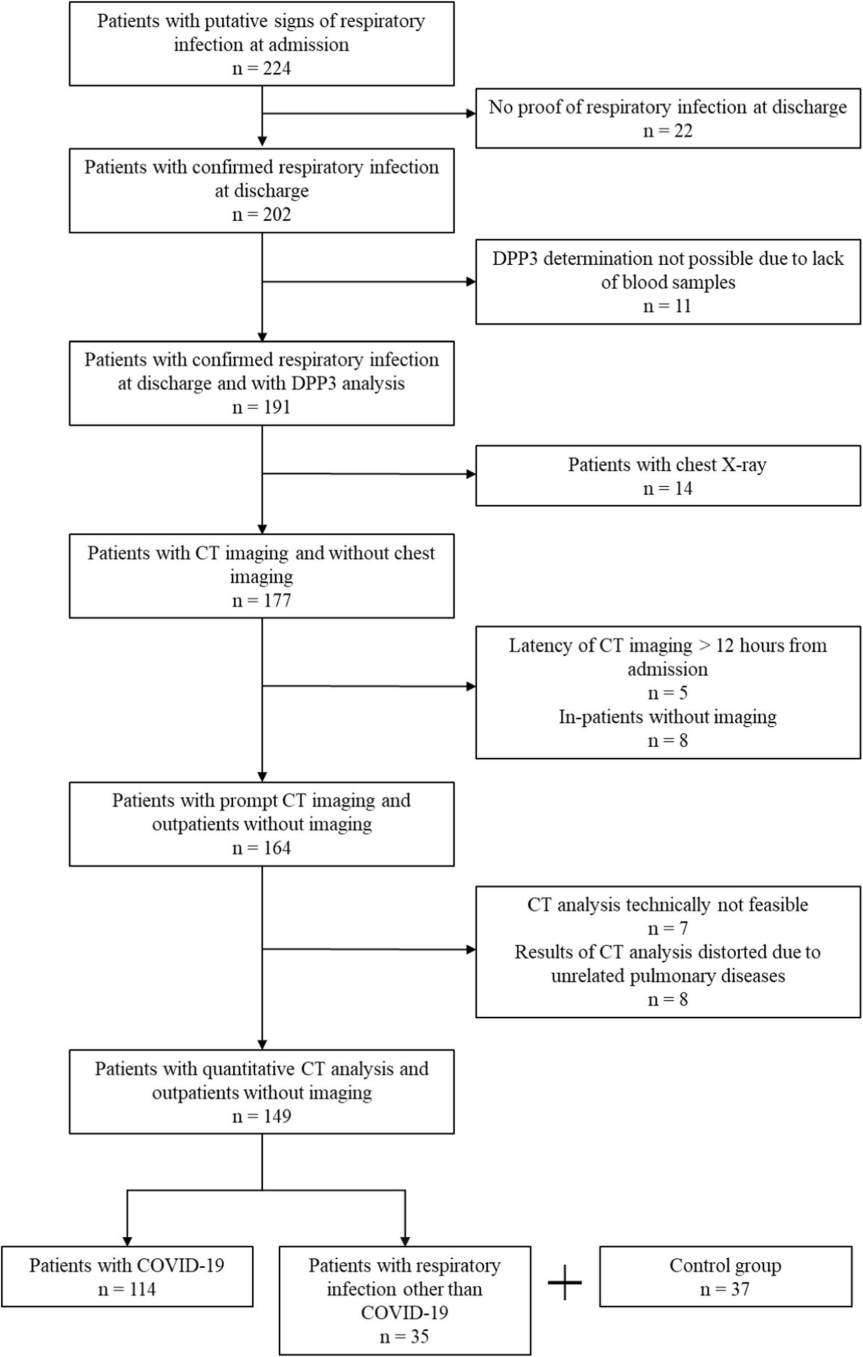
Flowchart on the study design

All patients were tested for SARS-CoV-2 via PCR (throat rinse water or nasopharyngeal swab). Depending on the PCR result, patients were divided into two groups: patients positive for SARS-CoV-2 were put in the COVID-19 group and patients negative for SARS-CoV-2 were put in the non-COVID-19 group. On top, patients without signs of respiratory infection were chosen as a healthy control group (in the following also referred to as control group). The patients of the healthy control group did not show any signs of an infection, relevant acute disease, or relevant pre-existing disease such as chronic pulmonary diseases, chronic kidney disease, or chronic cardiac disease. They presented themselves with psychogenic or musculoskeletal chest pain at the emergency department and their initial inclusion was conducted according to Schlossbauer et al. [27]. The flowchart on the study design can be seen in Fig. 1.

Baseline characteristics such as clinical examination findings, pre-existing diseases, vital parameters, and medication were gathered at inclusion. The National Early Warning Score 2 (NEWS-2) was used to determine the degree of illness of the patients included.

### Blood sampling and biochemical analysis

At the presentation to the emergency department, blood samples were collected to assess biomarker levels. EDTA plasma samples were frozen at − 80 °C and DPP3 was measured by 4TEEN4 Pharmaceuticals (Hennigsdorf, Germany) using the immunoluminometric assay sphingotest® DPP3 (SphingoTec GmbH, Hennigsdorf, Germany) as described previously [4]. Based on the manufacturer’s instruction for use, the 97.5th percentile for sphingotest® DPP3 in healthy adult subjects is 22 ng/mL (90% CI 18–34 ng/mL).

The assay is a microplate-based immunoluminometric assay that uses two antibodies against two different epitopes of DPP3 (the tracer antibody has a luminescent label while the capture antibody is linked to the microplate). Together with DPP3, the antibodies formed sandwich complexes on the microplate’s surface. The degree of luminescence is directly proportional to the concentration of DPP3 in the sample. Briefly, blood samples and kit components were defrosted. Following the manufacturer's instructions, 20 μL each of patient samples, controls, and standards were pipetted in the microtiter plate. 200 μL of the tracer antibody was added to the microtiter plate wells. The microplate was taped with black assay foil and incubated at room temperature (18–30 °C) for three hours (± 15 min) under shaking (600 rpm). Following this, the incubation mixture was removed from the wells, which were then repeatedly (five times) washed using the provided washing solution. Using sphingotest^®^ Lightning Reagents and Measurement Protocol, the luminescence signal of each microplate well was determined.

### Quantitative CT analysis

If necessary from a clinical perspective, patients received CT chest imaging. In case CT imaging was available, artificial intelligence software (syngo.via CT Pneumonia Analysis prototype by Siemens Healthineers, Munich, Germany) was used to quantify pulmonary infiltrates. The software detected and quantified hyperdense areas of the lung (ground glass opacities, consolidations, atelectasis, thickened pulmonary interstitial structures, reticular pattern, crazy paving pattern, fibrosis, and nodules) based on high-quality axial CT data with slice thicknesses up to 5 mm maximum, according to local standards of University Hospital Regensburg. Based on 3D segmentations of lesions, lungs, and lobes, the AI algorithm could determine the relative (“percentage of opacities”) and absolute volume of the lungs affected by opacities. The results provided by the software were validated in cooperation with expert radiologists from the Department of Radiology, University Hospital Regensburg. In case of discrepancy (results of the software distorted due to unrelated pulmonary diseases), the patients were excluded from the study (n = 8). The median of infiltrate was used to distinguish between patients with infiltrate above the median (55 COVID-19 and nine non-COVID-19) and patients with infiltrate below the median (47 COVID-19 and 19 non-COVID-19).

### Statistics

The Kolmogorov-Smirnoff test was used to test the variables for normal distribution. Regarding continuous variables, Student’s t-test was performed in normal distribution (mean, standard deviation) and Mann–Whitney-U test was performed in skewed distribution (median, interquartile range). Regarding binary variables, the Chi-square test or Fisher’s exact test was performed. The Spearman coefficient was used for correlation analysis. Besides, ROC curves and AUC values were determined. Furthermore, binary logistic regression analysis was performed regarding COVID-19 status and multiple linear regression analysis was performed regarding pulmonary infiltrates. Adjustment analyses have been performed concerning the potential confounding variables age, comorbidities (obesity, chronic pulmonary disease, and chronic kidney disease), and severe course of disease (intensive care and/or exitus). SPSS 25 (SPSS Inc., Chicago, Illinois) was used for statistical analysis.

## Results

### Study population

Clinical characteristics are shown in Table 1. 149 patients were included in the current study. Of these, 114 were SARS-CoV-2 positive and 35 suffered from respiratory infections other than COVID-19. The mean age was not significantly different between COVID-19 and non-COVID-19 (55.5 vs 55.6 years, *p* = n.s.). Patients were predominantly male (54.4% in COVID-19, 62.9% in non-COVID-19). Non-COVID-19 patients presented more often with coronary artery disease, chronic heart failure, COPD, and chronic kidney disease than COVID-19 patients (each *p* < 0.05). COVID-19 patients suffered more often from fatigue, anosmia, and dysgeusia than non-COVID-19 patients (each *p* < 0.05). Regarding long-term medication, diuretics were more often prescribed in non-COVID-19 patients than in COVID-19 patients (*p* < 0.05). Regarding the acute treatment, antibiotics were used significantly more often in non-COVID-19 than in COVID-19 patients (*p* < 0.05), whereas COVID-19 patients received significantly more frequent glucocorticoids and remdesivir (each *p* < 0.001). Concerning disease progression, congestive heart failure occurred significantly more often in non-COVID-19 than in COVID-19 patients (*p* < 0.05).

**Table 1:** Baseline data

	Respiratory patients (n = 149)	Control group (n = 37)	*p* value (respiratory patients vs control group)	COVID-19 (n = 114)	Non-COVID-19 (n = 35)	*p* value (COVID-19 vs non-COVID-19)
*Baseline characteristics*						
Age^f^ (y)	55.5 ± 16.4	45.2 ± 14.2	< 0.001^b^	55.5 ± 15.9	55.6 ± 18.3	0.97^b^
Sex, male, n (%)	84 (56.4)	23 (62.2)	0.52^c^	62 (54.4)	22 (62.9)	0.38^c^
Mortality, n (%)	12 (8.1)	–	–	10 (8.8)	2 (5.7)	0.56^c^
Intensive care unit, n (%)	24 (16.1)	–	–	19 (16.7)	5 (14.3)	0.74^c^
Hospitalisation, n (%)	120 (80.5)	–	–	96 (84.2)	24 (68.6)	0.052^d^
CT scan, n (%)	130 (87.2)	–	–	102 (89.5)	28 (80.0)	0.15^d^
CT scan with CM, n (%)	49 (32.9)	–	–	42 (36.8)	7 (20.0)	0.064^c^
*Symptoms*						
Cough, n (%)	99 (66.4)	–	–	75 (65.8)	24 (68.6)	0.76^c^
Fever, n (%)	88 (59.1)	–	–	65 (57.0)	23 (65.7)	0.36^c^
Dyspnea, n (%)	96 (64.4)	–	–	70 (61.4)	26 (74.3)	0.16^c^
Fatigue, n (%)	117 (78.5)	–	–	95 (83.3)	22 (62.9)	0.010^c^
Anosmia, n (%)	20 (13.4)	–	–	20 (17.5)	0 (0.0)	0.004^d^
Dysgeusia, n (%)	45 (30.2)	–	–	42 (36.8)	3 (8.6)	0.001^d^
*Pre-existing diseases*						
Arterial hypertension, n (%)	62 (41.6)	7 (18.9)	0.011^c^	44 (38.6)	18 (51.4)	0.18^c^
Diabetes mellitus, n (%)	27 (18.1)	0 (0.0)	0.003^d^	19 (16.7)	8 (22.9)	0.41^c^
Obesity, n (%)	41 (27.5)	5 (13.5)	0.077^c^	31 (27.2)	10 (28.6)	0.87^c^
Coronary artery disease, n (%)	19 (12.8)	0 (0.0)	0.016^d^	7 (6.1)	12 (34.3)	0.0^d^
Chronic heart failure, n (%)	9 (6.0)	0 (0.0)	0.21^d^	3 (2.6)	6 (17.1)	0.006^d^
COPD, n (%)	6 (4.0)	0 (0.0)	0.60^d^	1 (0.9)	5 (14.3)	0.003^d^
Asthma, n (%)	11 (7.4)	0 (0.0)	0.13^d^	8 (7.0)	3 (8.6)	0.72^d^
Chronic kidney disease, n (%)	22 (14.8)	0 (0.0)	0.009^d^	12 (10.5)	10 (28.6)	0.008^c^
*Physical markers*						
Respiratory rate^e^ (/min)	21 (17–26)	–	–	21 (17–26)	20 (18–24)	0.85^a^
Heart rate^f^ (b.p.m.)	88 ± 17	71 ± 11	< 0.001^b^	88 ± 15	90 ± 21	0.50^b^
Systolic blood pressure^f^ (mmHg)	130 ± 19	134 ± 15	0.25^b^	130 ± 18	132 ± 23	0.58^b^
Diastolic blood pressure^f^ (mmHg)	81 ± 13	83 ± 11	0.26^b^	81 ± 13	81 ± 14	0.96^b^
NEWS2^e^	4 (2–6)	–	–	4 (1.75–6)	3 (2–6)	0.73^a^
*Long-term medication*						
Immunosuppressants, n (%)	17 (11.4)	–	–	10 (8.8)	7 (20.0)	0.12^d^
Steroids, n (%)	11 (7.4)	–	–	6 (5.3)	5 (14.3)	0.13^d^
MMF, n (%)	5 (3.4)	–	–	4 (3.5)	1 (2.9)	1.00^d^
Tacrolimus, n (%)	4 (2.7)	–	–	3 (2.6)	1 (2.9)	1.00^d^
Biologicals, n (%)	3 (2.0)	–	–	2 (1.8)	1 (2.9)	0.56^d^
Azathioprine, n (%)	1 (0.7)	–	–	0 (0.0)	1 (2.9)	0.24^d^
Methotrexate, n (%)	2 (1.3)	–	–	1 (0.9)	1 (2.9)	0.42^d^
Cyclosporine A, n (%)	1 (0.7)	–	–	0 (0.0)	1 (2.9)	0.24^d^
ASS, n (%)	23 (15.4)	1 (2.7)	0.052^d^	16 (14.0)	7 (20.0)	0.39^c^
ACE-/AT-1-inhibitors, n (%)	42 (28.2)	5 (13.5)	0.066^c^	32 (28.1)	10 (28.6)	0.95^c^
Beta-blockers, n (%)	43 (28.9)	3 (8.1)	0.010^d^	31 (27.2)	12 (34.3)	0.42^c^
Diuretics, n (%)	31 (20.8)	3 (8.1)	0.096^d^	19 (16.7)	12 (34.3)	0.03^c^
Statins, n (%)	27 (18.1)	1 (2.7)	0.019^d^	17 (14.9)	10 (28.6)	0.07^c^
Insulin, n (%)	12 (8.1)	0 (0.0)	0.13^d^	8 (7.0)	4 (11.4)	0.48^d^
Metformin, n (%)	13 (8.7)	0 (0.0)	0.075^d^	10 (8.8)	3 (8.6)	1.0^d^
*Treatment*						
Conventional oxygen therapy, n (%)	66 (44.3)	–	–	54 (47.4)	12 (34.3)	0.17^c^
High-flow oxygen therapy, n (%)	16 (10.7)	–	–	13 (11.4)	3 (8.6)	0.76^d^
Non-invasive ventilation therapy, n (%)	13 (8.7)	–	–	12 (10.5)	1 (2.9)	0.30^d^
Invasive ventilation therapy, n (%)	16 (10.7)	–	–	15 (13.2)	1 (2.9)	0.12^d^
ECMO, n (%)	2 (1.3)	–	–	2 (1.8)	0 (0)	1.00^d^
Catecholamines, n (%)	14 (9.4)	–	–	10 (8.8)	4 (11.4)	0.74^d^
Antibiotic therapy, n (%)	64 (43.0)	–	–	43 (37.7)	21 (60.0)	0.02^c^
Glucocorticoids, n (%)	51 (34.2)	–	–	50 (43.9)	1 (2.9)	< 0.001^d^
Remdesivir, n (%)	32 (21.5)	–	–	32 (28.1)	0 (0.0)	< 0.001^d^
*Disease progression*						
Acute kidney injury, n (%)	27 (18.1)	–	–	19 (16.7)	8 (22.9)	0.41^c^
Congestive heart failure, n (%)	9 (6.0)	–	–	4 (3.5)	5 (14.3)	0.03^d^
Cerebral ischemia, n (%)	0 (0.0)	–	–	0 (0.0)	0 (0.0)	–
Cerebral hemorrhage, n (%)	1 (0.67)	–	–	1 (0.88)	0 (0.0)	1.00^d^
Myocardial infarction, n (%)	0 (0.0)	–	–	0 (0.0)	0 (0.0)	–
Pulmonary embolism, n (%)	6 (4.0)	–	–	5 (4.4)	1 (2.9)	1.00^d^
*Biomarkers*						
CT infiltrate^e^, %	14.45 (3.7–34.0)	–	–	17.0 (5.8–42.1)	4.8 (0.2–16.4)	< 0.001^a^
DPP3^e^ (ng/ml)	18.8 (13.4–28.9)	10.6 (7.9–15.4)	< 0.001^a^	20.9 (14.9–32.1)	13.8 (9.1–19.7)	< 0.001^a^
IL-6^e^ (pg/ml)	36.2 (15.2–76.1)	2.2 (1.4–4.0)	< 0.001^a^	34.3 (16.4–70.5)	36.4 (11.3–200.8)	0.59^a^
CRP^e^ (mg/dl)	48.2 (19.6–93.5)	2.9 (2.9–3.1)	< 0.001^a^	44.5 (20.3–93.4)	50.4 (8.8–93.8)	0.64^a^
Leucocytes^e^ (/nl)	6.65 (4.47–9.07)	7.39 (5.92–8.86)	0.19^a^	5.74 (4.02–7.87)	10.42 (7.18–13.20)	< 0.001^a^

^a^Mann–Whitney-U-test

^b^Student’s t-test

^c^Chi-squared test (two-tailed)

^d^Fisher’s exact test (two-tailed)

^e^Median (interquartile range)

^f^Mean ± standard deviation

Thirty-seven patients without signs of respiratory infection or relevant acute disease (presentation with psychogenic or musculoskeletal chest pain at the emergency room) were used as a healthy control group. The healthy control patients were significantly younger than the respiratory patients (*p* < 0.001, mean age 45.2 years). Furthermore, they suffered significantly less often from arterial hypertension (*p* < 0.05), diabetes mellitus (*p* < 0.05), coronary artery disease (*p* < 0.05), and chronic kidney disease (*p* < 0.05). Beta-blockers (*p* < 0.05) and statins (*p* < 0.05) were prescribed significantly less in the healthy control patients.

Radiological findings of patients with CT imaging (n = 130, thereof 102 COVID-19 and 28 non-COVID-19) are shown in Table 2. Pulmonary infiltrates were detected significantly more frequent in COVID-19 patients as compared to non-COVID-19 (*p* < 0.05). In addition, the median percentage of infiltrates was significantly higher in COVID-19 than in non-COVID-19 patients (*p* < 0.001). Moreover, ground glass opacities (*p* < 0.001), atelectasis and pleural effusion (each *p* < 0.05) were significantly more often in the COVID-19 group than in the non-COVID-19 group.

**Table 2:** Radiological findings of patients with CT imaging

	COVID-19 (n = 102)	Non-COVID-19 (n = 28)	*p* value (COVID-19 vs non-COVID-19)
Pulmonary infiltrates, n (%)	97 (95.1)	21 (75.0)	< 0.05^c^
Median percentage of infiltrates^d^, %	17.0 (5.8–42.1)	4.8 (0.2–16.4)	< 0.001^a^
Ground glass opacities, n (%)	93 (91.2)	17 (60.7)	< 0.001^c^
Consolidations, n (%)	56 (54.9)	15 (53.6)	0.90^b^
Atelectasis, n (%)	7 (6.9)	6 (21.4)	< 0.05^c^
Pleural effusion, n (%)	9 (8.8)	9 (32.1)	< 0.05^c^
Pulmonary venous congestion, n (%)	4 (3.9)	4 (14.3)	0.065^c^
Pulmonary embolism, n (%)	5 (4.9)	1 (3.6)	1.00^c^

^a^Mann–Whitney-U-test

^b^Chi-squared test (two-tailed)

^c^Fisher’s exact test (two-tailed)

^d^Median (interquartile range)

### Detection of COVID-19

DPP3 showed a significant and positive correlation with IL-6 (ρ = 0.50, *p* < 0.001), CRP (ρ = 0.46, *p* < 0.001), and leucocytes (ρ = 0.20, *p* < 0.05) in COVID-19 patients. In non-COVID-19, DPP3 was significantly correlated with CRP (ρ = 0.35, *p* < 0.05), as opposed to IL-6 and leucocytes (each *p* = n.s.). In both groups, IL-6 was significantly correlated with CRP (COVID-19: ρ = 0.73, *p* < 0.001; non-COVID-19: ρ = 0.70, *p* < 0.001) and leucocytes (COVID-19: ρ = 0.39, *p* < 0.001; non-COVID-19: ρ = 0.57, *p* < 0.001).

**Fig. 2:**
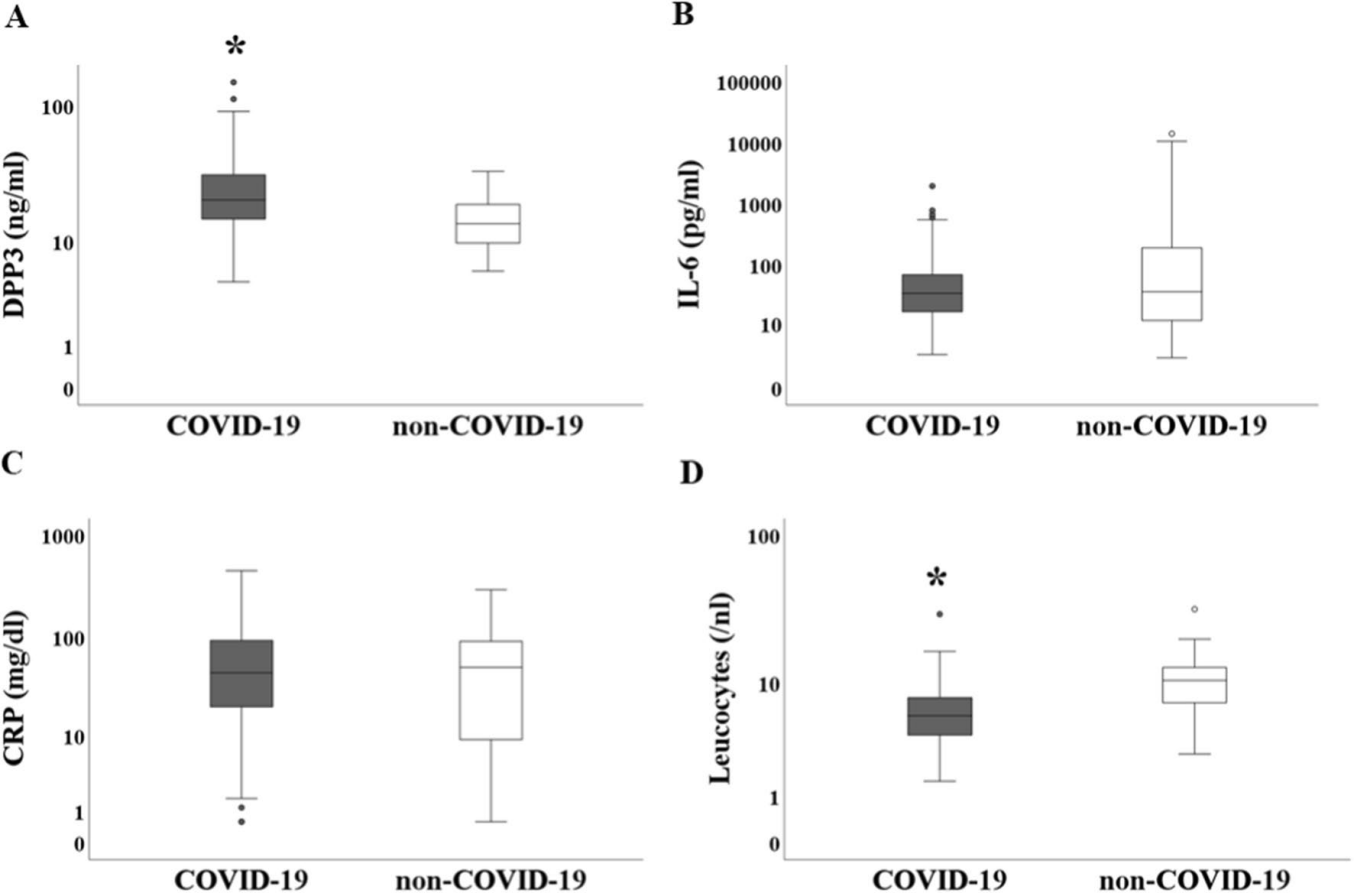
Boxplots showing the biomarkers DPP3, IL-6, CRP and leucocytes in COVID-19 (gray colour) as compared to non-COVID-19 (white colour). COVID-19 n = 114, non-COVID-19 n = 35. **A** DPP3 was significantly elevated in COVID-19 patients as compared to patients with a respiratory infection other than SARS-CoV-2 (*p* < 0.001). **B** IL-6 showed no significant difference between COVID-19 and non-COVID-19 (*p* = n.s.). **C** CRP did not differ significantly between COVID-19 and non-COVID-19 (*p* = n.s.). **D** Leucocytes were significantly higher in the non-COVID-19 cohort as compared to COVID-19 (*p* < 0.001). *p < 0.001. Logarithmic display was used in the y-axis

DPP3 was significantly increased in COVID-19 patients compared to those without SARS-CoV-2 (*p* < 0.001), opposite to IL-6 and CRP (each *p* = n.s., Fig. 2, Table 3). In addition, leucocytes were significantly higher in non-COVID-19 than in COVID-19 (*p* < 0.001, Fig. 2, Table 3).

**Table 3:** Median and 95% confidence interval of biomarkers in COVID-19 versus non-COVID-19

	COVID-19^a^	Non-COVID-19^a^	*p* value^b^
DPP3 (ng/ml)	20.85 (18.30–24.80)	13.80 (11.30–17.65)	< 0.001
IL-6 (pg/ml)	34.25 (27.30–43.90)	36.40 (19.15–63.15)	n.s
CRP (mg/dl)	44.45 (33.61–64.80)	50.40 (36.40–68.70)	n.s
Leucocytes (/nl)	5.74 (5.35–6.13)	10.42 (7.33–11.75)	< 0.001

^a^Median (95% confidence interval)

^b^Mann–Whitney-U-test

**Fig. 3:**
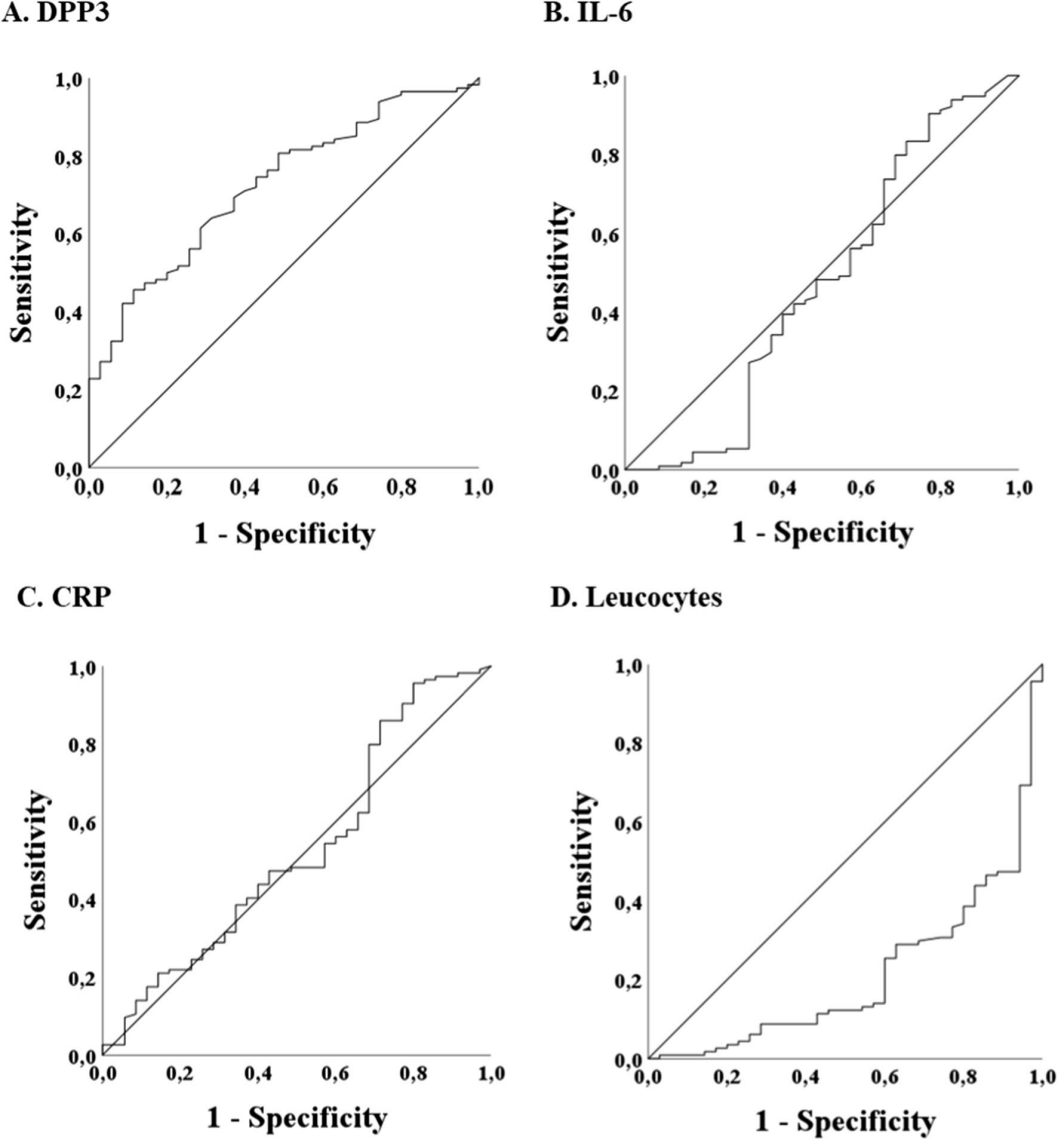
ROC analysis regarding the detection of COVID-19 infection. COVID-19 n = 114, non-COVID-19 n = 35. **A** DPP3 was able to detect a COVID-19 infection with an AUC of 0.72 (*p* < 0.001, cut-off 16.4 ng/ml, sensitivity 71.7%, specificity 60.0%). **B** IL-6 was not able to detect a COVID-19 infection (*p* = n.s., AUC = 0.47). **C** CRP was not able to detect a COVID-19 infection (*p* = n.s., AUC = 0.53). **D** Leucocytes showed an AUC of 0.21 regarding the detection of a COVID-19 infection (*p* < 0.001)

In ROC analysis regarding the detection of COVID-19, DPP3 showed an AUC of 0.72 (*p* < 0.001; cut-off 16.4 ng/ml: sensitivity 71.7%, specificity 60.0%), opposite to IL-6 (AUC = 0.47, *p* = n.s.) and CRP (AUC = 0.53, *p* = n.s.; Fig. 3, Table 4). Regarding the detection of non-COVID-19 patients, leucocytes showed an AUC of 0.79 (*p* < 0.001, cut-off 7.17/nl: sensitivity 77.1%, specificity 69.3%).

**Table 4:** ROC analysis: detection of COVID-19 compared to non-COVID-19

	*p* value^a^	AUC^b^	95% confidence interval^b^	Cut-off^b^	Sensitivity^b^	Specificity^b^
DPP3	< 0.001	0.72	0.64–0.81	16.4 ng/ml	71.7%	60.0%
IL-6	n.s	0.47	0.34–0.60	–	–	–
CRP	n.s	0.53	0.41–0.64	–	–	–
Leucocytes^c^	< 0.001	0.21	0.13–0.30	–	–	–

^a^Mann–Whitney-U-test

^b^ROC analysis

^c^Leucocytes regarding the detection of non-COVID-19: cut-off 7.17/nl, sensitivity 77.1%, specificity 69.3%

Regarding detection of COVID-19, multivariate stepwise binary logistic regression analyses adjusted for the potential confounding variables were performed separately for each DPP3 and leucocytes (Table 5). On the one hand, DPP3, no chronic pulmonary disease and no chronic kidney disease were identified as significant and independent predictors for COVID-19 infection (each *p* < 0.05), opposite to age, obesity, and severe course of disease (each *p* = n.s.). On the other hand, lower leucocytes counts (*p* < 0.001), no chronic pulmonary disease, no chronic kidney disease, and severe course of disease (each *p* < 0.05) were identified as significant and independent predictors for COVID-19 infection, opposite to age and obesity (each *p* = n.s.).

**Table 5:** DPP3 and leucocytes: Multivariate stepwise binary logistic regression analysis adjusted for the potential confounding variables regarding COVID-19 infection

	DPP3			Leucocytes		
	*p* value	Odds ratio	95% CI	*p* value	Odds ratio	95% CI
Biomarker	0.001	1.10	1.04–1.16	< 0.001	0.78	0.69–0.87
Age	n.s	1.00	0.97–1.03	n.s	1.01	0.98–1.04
Obesity	n.s	0.93	0.36–2.43	n.s	1.13	0.41–3.13
Chronic pulmonary disease	< 0.05	0.22	0.07–0.74	< 0.05	0.21	0.06–0.73
Chronic kidney disease	< 0.05	0.28	0.10–0.79	< 0.05	0.23	0.07–0.72
Severe course of disease	n.s	0.90	0.23–3.48	< 0.05	4.87	1.10–21.6

Regarding binary logistic regression analysis, higher DPP3 concentrations (OR = 1.12, *p* < 0.001) and lower leucocytes counts (OR = 0.76, *p* < 0.001) were identified as significant and independent predictors of SARS-CoV-2 infection, as opposed to IL-6 and CRP (each *p* = n.s., Table 6).

**Table 6:** Binary logistic regression analysis for COVID-19 infection

	*p* value	Odds ratio	95% confidence interval
DPP3	< 0.001	1.12	1.06–1.19
IL-6	n.s	1.00	1.00–1.00
CRP	n.s	1.01	1.00–1.02
Leucocytes	< 0.001	0.76	0.68–0.85

### Pulmonary infiltrates

89.5% of COVID-19 patients and 80.0% of non-COVID-19 patients received CT imaging (*p* = n.s.; 19 outpatients (12 COVID-19 and 7 non-COVID-19) did not receive CT imaging). The overall patient population showed a median infiltrate of 14.45% (IQR 3.7–34.0%). Among them, the extent of pulmonary infiltrates was significantly more distinct in COVID-19 patients (17.0%, IQR 5.8–42.1%) compared to non-COVID-19 patients (4.8%, IQR 0.2–16.4%; *p* < 0.001).

DPP3 (ρ = 0.46, *p* < 0.001), CRP (ρ = 0.67, *p* < 0.001), and leucocytes (ρ = 0.39, *p* < 0.001) were significantly correlated with pulmonary infiltrates in COVID-19 patients, as opposed to non-COVID-19 patients (each *p* = n.s.). IL-6 showed in both groups a significant correlation with pulmonary infiltrates (COVID-19: ρ = 0.65, *p* < 0.001; non-COVID-19: ρ = 0.60, *p* < 0.05).

**Fig. 4:**
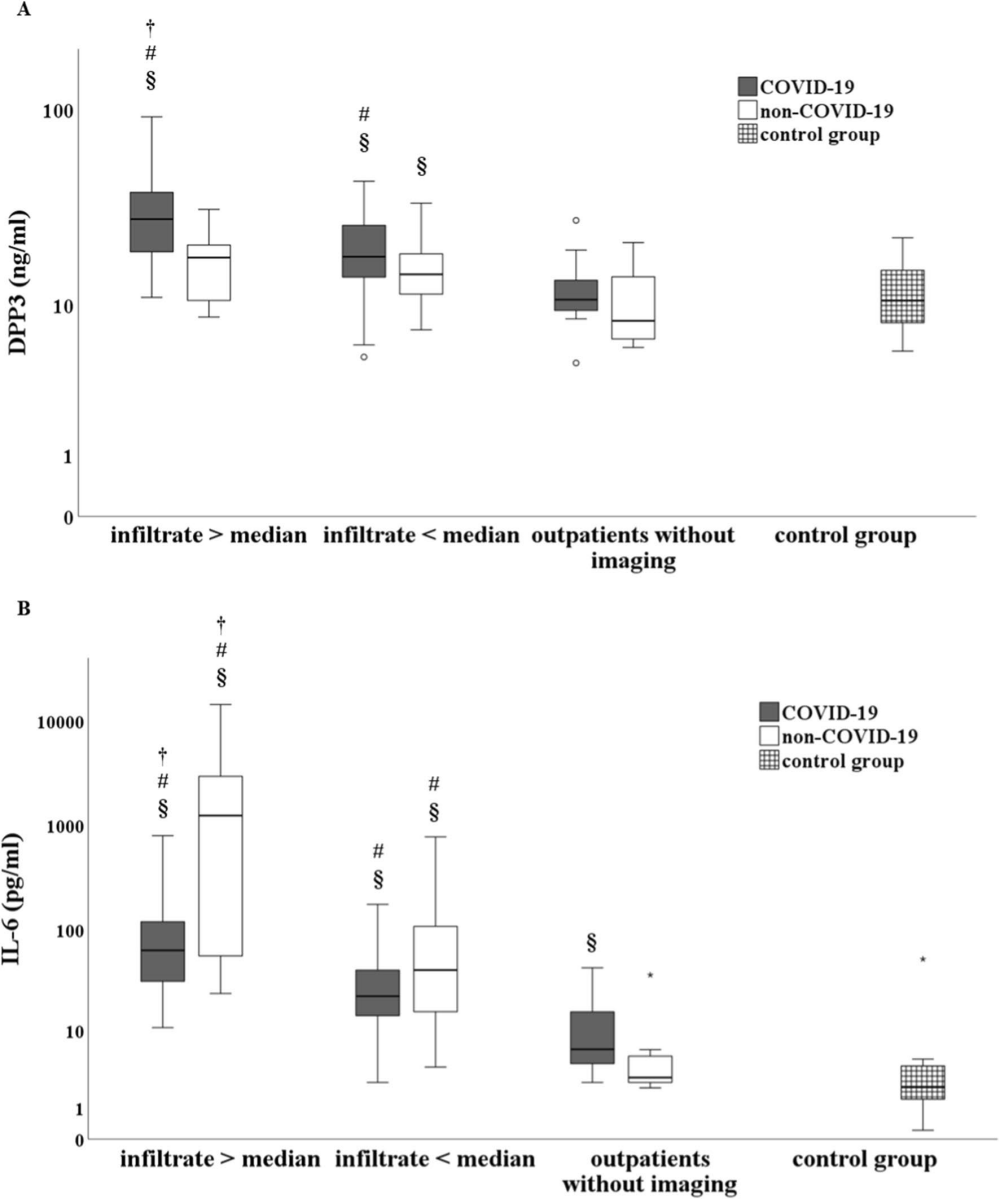
Boxplots showing DPP3 and IL-6 regarding pulmonary infiltrates in COVID-19 (gray colour) and in non-COVID-19 (white colour). Median of infiltrate = 14.45%. Infiltrate > median: COVID-19 n = 55 and non-COVID-19 n = 9. Infiltrate < median: COVID-19 n = 47 and non-COVID-19 n = 19. Outpatients without imaging: COVID-19 n = 12 and non-COVID-19 n = 7. Control group: n = 37. **A** DPP3 was significantly elevated in COVID-19 patients with infiltrate above median as compared to infiltrate below median (*p* < 0.001), outpatients without imaging (*p* < 0.001) and control group (*p* < 0.001), opposite to non-COVID-19 (each *p* = n.s.). **B** IL-6 showed a significant and stepwise increase between the subgroups (infiltrate above median, infiltrate below median, outpatients without imaging) both in COVID-19 and in non-COVID-19 (each *p* < 0.05). † *p* < 0.05 compared to infiltrate < median. # *p* < 0.05 compared to outpatients without imaging. § *p* < 0.05 compared to control group. Logarithmic display was used in the y-axis

DPP3, IL-6, CRP, and leucocytes were significantly elevated in COVID-19 patients with infiltrate above the median compared to patients with infiltrate below the median (each *p* < 0.05) and outpatients without imaging (each *p* < 0.05). However, only IL-6 showed a significant and stepwise increase between these subgroups in non-COVID-19 patients (each *p* < 0.05), opposite to DPP3 and leucocytes (each *p* = n.s.). Boxplots are shown in Fig. 4.

In COVID-19 patients, ROC analysis regarding the detection of infiltrate above the median compared to infiltrate below the median showed satisfying predictive values for CRP (AUC = 0.82, *p* < 0.001) and IL-6 (AUC = 0.78, *p* < 0.001; Table 7). AUC values for DPP3 (AUC = 0.70, *p* < 0.05; cut off 18.9 ng/ml: sensitivity 78.2%, specificity 59.6%) and leucocytes (AUC = 0.67, *p* < 0.05) were significantly lower compared to CRP (each *p* < 0.05).

**Table 7:** ROC analysis: detection of infiltrate above median compared to infiltrate below median in COVID-19

	*p* value^a^	AUC^b^	95% confidence interval^b^	Cut-off^b^	Sensitivity^b^ (%)	Specificity^b^ (%)
DPP3	< 0.001	0.70	0.59–0.80	18.9 ng/ml	78.2	59.6
IL-6	< 0.001	0.78	0.69–0.87	34.3 pg/ml	70.9	66.0
CRP	< 0.001	0.82	0.74–0.90	43.2 mg/dl	80.0	70.2
Leucocytes	< 0.05	0.67	0.57–0.78	5.7 /nl	67.3	61.7

^a^Mann–Whitney-U-test

^b^ROC analysis

Regarding detection of infiltrate above median compared to infiltrate below median in COVID-19, multivariate stepwise binary logistic regression analyses adjusted for the potential confounding variables were performed separately for each DPP3, IL-6, CRP, and leucocytes (Table 8). Firstly, severe course of disease was significantly and independently associated with infiltrate above the median in COVID-19 (*p* < 0.05), opposite to DPP3, age, obesity, chronic pulmonary disease, and chronic kidney disease (each *p* = n.s.). Secondly, IL-6 was identified as a significant and independent predictor for infiltrate above the median in COVID-19 (*p* < 0.001), opposite to age, obesity, chronic pulmonary disease, chronic kidney disease, and severe course of disease (each *p* = n.s.). Thirdly, CRP was identified as a significant and independent predictor for infiltrate above the median in COVID-19 (*p* < 0.001), opposite to age, obesity, chronic pulmonary disease, chronic kidney disease, and severe course of disease (each *p* = n.s.). Fourthly, leucocytes and severe course of disease were significantly and independently associated with infiltrate above the median in COVID-19 (each *p* < 0.05), opposite to age, obesity, chronic pulmonary disease, and chronic kidney disease (each *p* = n.s).

**Table 8:** DPP3, IL-6, CRP, and leucocytes: multivariate stepwise binary logistic regression analysis adjusted for the potential confounding variables regarding detection of infiltrate above median compared to infiltrate below median in COVID-19

	DPP3			IL-6			CRP			Leucocytes		
	*p* value	Odds ratio	95% CI	*p* value	Odds ratio	95% CI	*p* value	Odds ratio	95% CI	*p* value	Odds ratio	95% CI
Biomarker	n.s	1.01	0.99–1.03	< 0.001	1.03	1.01–1.04	< 0.001	1.03	1.01–1.04	< 0.05	1.17	1.003–1.37
Age	n.s	1.01	0.98–1.04	n.s	1.00	0.97–1.04	n.s	1.01	0.97–1.04	n.s	1.01	0.98–1.04
Obesity	n.s	1.37	0.51–3.67	n.s	1.23	0.41–3.69	n.s	1.05	0.34–3.26	n.s	1.34	0.49–3.66
Chronic pulmonary disease	n.s	0.63	0.14–2.88	n.s	0.17	0.02–1.57	n.s	0.58	0.09–3.52	n.s	0.67	0.15–2.94
Chronic kidney disease	n.s	1.18	0.28–4.94	n.s	1.48	0.29–7.48	n.s	2.08	0.43–10.1	n.s	1.16	0.27–4.90
Severe course of disease	< 0.05	7.14	1.95–26.1	n.s	1.70	0.35–8.30	n.s	2.39	0.54–10.5	< 0.05	5.67	1.50–21.4

Regarding multiple linear regression analysis in COVID-19, CRP, IL-6, and leucocytes (each *p* < 0.05) were associated with the degree of pulmonary infiltrates, as opposed to DPP3 (*p* = n.s., Table 9).

**Table 9:** Multiple linear regression analysis for pulmonary infiltrates in COVID-19

	*p* value	Regression coefficient	95% confidence interval
DPP3 (ng/ml)	n.s	0.034	− 0.13–0.20
IL-6 (pg/ml)	< 0.05	0.019	0.002–0.035
CRP (mg/dl)	< 0.001	0.103	0.052–0.153
Leucocytes (/nl)	< 0.05	1.214	0.18–2.25

Regarding the percentage of pulmonary infiltrates in COVID-19, stepwise multiple linear regression analyses adjusted for the potential confounding variables were performed separately for each IL-6, CRP, and leucocytes (Table 10). Firstly, IL-6, obesity (each *p* < 0.05), and severe course of disease (*p* < 0.001) were significantly and independently associated with the percentage of pulmonary infiltrates in COVID-19, opposite to age, chronic pulmonary disease, and chronic kidney disease (each *p* = n.s.). Secondly, CRP, severe course of disease (each *p* < 0.001), and obesity (*p* < 0.05) were significantly and independently associated with the percentage of pulmonary infiltrates in COVID-19, opposite to age, chronic pulmonary disease, and chronic kidney disease (each *p* = n.s.). Thirdly, leucocytes and severe course of disease (each *p* < 0.001) were significantly and independently associated with the percentage of pulmonary infiltrates in COVID-19, opposite to age, obesity, chronic pulmonary disease, and chronic kidney disease (each *p* = n.s.).

**Table 10:** IL-6, CRP, and leucocytes: stepwise multiple linear regression analysis adjusted for the potential confounding variables regarding the percentage of pulmonary infiltrates in COVID-19

	IL-6			CRP			Leucocytes		
	*p* value	Odds ratio	95% CI	*p* value	Odds ratio	95% CI	*p* value	Odds ratio	95% CI
Biomarker	< 0.05	0.023	0.006–0.04	< 0.001	0.098	0.05–0.15	< 0.001	1.83	0.94–2.72
Age	n.s	0.05	− 0.19–0.29	n.s	0.02	− 0.21–0.25	n.s	0.038	− 0.19–0.27
Obesity	< 0.05	8.58	0.76–16.4	< 0.05	7.48	0.78–14.9	n.s	7.34	− 0.07–14.7
Chronic pulmonary disease	n.s	− 5.78	− 17.9–6.31	n.s	− 3.45	− 15.0–8.10	n.s	− 1.99	− 13.6–9.58
Chronic kidney disease	n.s	− 3.95	− 16.1–8.15	n.s	− 1.27	− 12.2–9.70	n.s	2.09	− 8.73–12.9
Severe course of disease	< 0.001	22.8	13.3–32.3	< 0.001	19.7	10.5–28.9	< 0.001	23.6	15.2–32.1

In non-COVID-19 patients, only IL-6 was able to distinguish infiltrate above the median from infiltrate below the median (AUC = 0.81, *p* < 0.05, cut-off 15.3 pg/ml: sensitivity 77.8%, specificity 63.2%), opposite to DPP3 (AUC = 0.56, *p* = n.s.), CRP (AUC = 0.47, *p* = n.s.) and leucocytes (AUC = 0.61, *p* = n.s.).

## Discussion

### Summary of results

The current study examined the predictive value of DPP3, IL-6, CRP, and leucocytes of COVID-19 infection and pulmonary infiltrates in respiratory patients. For the first time, DPP3 was shown to be a significant and independent predictor of COVID-19 infection, as opposed to IL-6, CRP, and leucocytes. Moreover, IL-6 was significantly and independently associated with the degree of pulmonary infiltrates in COVID-19 and was able to detect infiltrate above median in respiratory infections other than SARS-CoV-2.

### DPP3: a COVID-19 specific biomarker

DPP3 is a ubiquitous zinc-dependent aminopeptidase whose substrates are oligopeptides consisting of three to 10 residues. Its most essential substrates are angiotensins, enkephalins, and endomorphins, indicating its comprehensive physiological role in signal transduction, pain modulation, blood pressure regulation, and immunomodulation [2, 3]. So far, DPP3 has been described as a biomarker in sepsis, indicating severity and mortality [4, 5]. As well as in sepsis, hyperinflammation plays a key role in the pathophysiology of COVID-19 [28]. Consequently, DPP3 levels in the blood might increase due to inflammatory processes coming along with COVID-19 infection. In addition, DPP3 has been described as a biomarker with prognostic value for clinical outcomes in intensive care patients, cardiogenic shock, and severely ill burn patients [5–7, 29, 30]. In rodent models, inhibition of DPP3 via a monoclonal antibody was shown to restore cardiac function in sepsis and improve haemodynamics in heart failure [31, 32].

Physiologically, DPP3 hydrolyses angiotensin II, a vasoconstrictor and key effector in the renin–angiotensin–aldosterone system (RAAS) [3, 9]. Angiotensin II is converted by ACE2, a counter-regulatory enzyme to ACE [8]. Membrane-bound ACE2 is a functional receptor for SARS-CoV-2 to enter the host cell [10, 11]. Furthermore, previous studies have shown that SARS-CoV-2 binding ACE2 leads to the downregulation of ACE2 and, thereby, to an increase in angiotensin II, causing vasculopathy, coagulopathy, and inflammation [33, 34]. Hypothetically, DPP3 as a degrading enzyme of angiotensin II might rise in response. Moreover, DPP3 is suggested to be a marker for cell death, and DPP3 blood levels could potentially rise during inflammatory processes [5]. Thus, ACE2 may be a link between SARS-CoV-2 and DPP3, implying that DPP3 might have a role in the pathophysiology of COVID-19.

So far, the existing data on the role of DPP3 in COVID-19 is restricted to critically ill patients. Van Lier et al. showed that DPP3 is associated with poor clinical outcomes in intensive-care COVID-19 patients [12]. In their case series (n = 6), Heinicke et al. measured DPP3 levels in severe COVID-19 patients to assess a potential deficiency of serum Angiotensin II and evaluate the possible benefit of Angiotensin II administration. DPP3 was used as a decision guidance for therapeutic intervention in severely affected COVID-19 patients [13]. The current study examined DPP3 regarding its predictive value for the diagnosis of COVID-19 infection.

For the first time, this study evaluated the role of DPP3 in COVID-19 patients compared to a non-COVID-19 group in an emergency ambulance setting. In particular, patients with a negative PCR result for SARS-CoV-2 enabled comparison of the DPP3 levels to respiratory infections other than COVID-19. It was shown that DPP3 is a significant and independent predictor of SARS-CoV-2 infection. This might provide additional value in diagnostics of COVID-19 as well as help understand the pathophysiology of COVID-19.

Karami et al. identified a novel hub gene signature in respiratory cells infected by SARS-CoV-2 using weighted gene co-expression network analysis. Relevant biological pathways in COVID-19 (including among others the type I and IL-17 signaling pathway) were determined. The results contribute to a more profound knowledge of pathophysiology in COVID-19 paving the way for future research on biomarkers and treatment options for COVID-19 [26]. Similarly, the current study was designed to identify a suitable COVID-19 biomarker that may provide information on therapeutic options in the future. Karami et al. characterized transcriptional changes in respiratory cells to explore pivotal pathways in the pathophysiology of COVID-19. In contrast, in the current study an immunoluminometric assay was performed to quantify blood levels of DPP3. Karami et al. offered future treatment options in COVID-19. Similarly, the current study may indicate Procizumab, the specific antibody against DPP3, as an imaginable therapeutic option in the future. In accordance with Karami et al., applying methods to identify transcriptional changes and measuring its gene expression may be an additional way of assessing the implications of DPP3 in COVID-19 in the future. The intention may be to uncover biological processes and their key modules to elucidate prognostic markers and drug targets.

Further studies will be needed to evaluate whether and how DPP3 might be involved in COVID-19 pathophysiology. If this should be confirmed, the specific antibody against DPP3, Procizumab, may be examined regarding its therapeutic value [31].

As aforementioned, literature describes DPP3 as a biomarker in various diseases. Hence, comorbidities and other factors need to be considered that may affect DPP3 levels and its COVID-19 specificity. To estimate their influence and to minimize their impact, adjustment analyses have been performed to statistically account for the confounding variables age, obesity, chronic pulmonary disease, chronic kidney disease, and diseases severity (intensive care and/or exitus).

To determine DPP3 levels in the blood samples, the luminometric immunoassay was used. It has been described as a highly specific assay for the quantification of DPP3 with a robust performance [4]. In a further study, the enzyme capture activity assay can be performed in addition to provide even more robust data, strengthening the biomarker’s sensitivity and specificity [4].

In the multivariate regression analyses regarding COVID-19 infection, no chronic pulmonary disease and no chronic kidney disease were identified as significant and independent predictors of COVID-19 infection. This may be explained by the fact that in our study cohort the non-COVID-19 patients showed significantly more often COPD and chronic kidney disease as compared to the COVID-19 patients (see Baseline Table).

### IL-6: a predictor of pulmonary infiltrates

IL-6 is a pleiotropic cytokine released by various immune system cells and other tissues, regulating the local and systemic inflammatory response [35–37]. Via the membrane-bound IL-6 receptor expressed on hepatocytes, IL-6 triggers the synthesis of acute phase proteins in the liver, causing further inflammatory processes [38]. Due to its diverse places of action and complex signalling pathways, IL-6 plays a key role in host defence [39]. In particular, IL-6 has been described as crucial for the transition from innate to acquired immunity [40].

IL-6 has been evaluated as a biomarker in sepsis, septic shock, and community-acquired pneumonia [14–17, 41]. In pneumonia, IL-6 was shown to be associated with mortality and disease severity [42–44]. Furthermore, IL-6 has been described as a predictor of clinical outcomes in ARDS [45]. However, in an observational study comparing acute respiratory distress syndrome, severe pneumonia, and controls, IL-6 was not significantly different between the groups [46]. Thus, the existing data regarding the role of IL-6 in respiratory infectious disease is not entirely consistent. It particularly focuses on the predictive value of IL-6 to the clinical outcome parameters of mortality, intensive care unit treatment, and mechanical ventilation. The current study supports and extends the existing literature by showing that IL-6 is associated with the degree of pulmonary infiltrates in a diverse emergency department collective.

In COVID-19, IL-6 has been characterized as an indicator of cytokine release syndrome and thus a prognostic marker for the clinical outcome [18, 19]. In their retrospective study, Chen et al. examined the association of laboratory markers with lung infiltrates in COVID-19. Two separate physicians evaluated CT images and classified lung involvement semi-quantitatively. As a result, IL-6 was shown to be an independent predictor for lung injury in COVID-19 pneumonia [20]. The current study confirms and extends these findings by applying artificial intelligence software to quantify pulmonary infiltrates as objectively and exactly as possible. Moreover, a non-COVID-19 group was included to assess differences with other respiratory infections. In a series of retrospective studies, the pulmonary infiltrates of COVID-19 patients were quantified regarding their predictive value for the clinical outcome [21–25]. Instead, the current prospective study focussed on the predictive value of laboratory markers for the degree of lung infiltrates.

IL-6 antagonists have been described as therapeutic options in various rheumatic diseases such as rheumatoid arthritis and juvenile idiopathic arthritis [47, 48]. In COVID-19, anti-IL-6 receptor antibodies have been discussed as therapeutic tools to inhibit cytokine storms and improve clinical outcome [49, 50]. The current study affirms the role of IL-6 in respiratory patients with cytokine release syndrome and severe pulmonary lesions. Hence, IL-6 antagonists may be of therapeutic value to prevent the development of severe lung infiltrates and sepsis early and efficiently.

The artificial intelligence software used in the current study detects hyperdense areas of the lungs such as ground glass opacities and consolidations, which have repeatedly been described as the predominant imaging characteristics in COVID-19 [51–55]. However, it also detects other manifestations of COVID-19 that show elevated density in CT imaging such as atelectasis, thickened pulmonary interstitial structures (reticular pattern, crazy paving pattern), fibrosis, and nodules [51]. CT imaging manifestation of COVID-19 that either do not show elevated density (e.g., air bubble sign) or are located outside the lungs (e.g., lymphadenopathy and pericardial effusion) are consequently not detected by the artificial intelligence software. However, they occur rather rarely (e.g., lymphadenopathy in 4–8% of COVID-19 cases, pericardial effusion in 5–6% of COVID-19 cases) [51, 53, 55–57]. In addition, the aim of the study was to provide the degree of COVID-19 lung manifestations as accurate as possible rather than qualitatively describe possible forms of chest CT findings in COVID-19.

For the first time, this study evaluated IL-6 in a broad collective of respiratory patients—from upper respiratory tract infection to pneumonia to sepsis with ARDS—including COVID-19 and non-COVID-19 regarding the degree of lung infiltrates. Specifically, IL-6 was compared to DPP3 and the inflammatory markers CRP and leucocytes. Further studies will be necessary to determine if our data can be confirmed, particularly in a larger non-COVID-19 group. Additionally, further interventional studies will be needed to evaluate if anti-IL-6 antibodies may be an adequate therapeutical option.

### DPP3 and IL-6: additional value for diagnostics in COVID-19 and other respiratory infections

In the future, DPP3 might provide additional diagnostic information on respiratory patients in an emergency ambulance setting. Particularly in COVID-19, DPP3 may be used as a complementary biomarker combining detection and prognostic value. Necessary hygienic and therapeutic measures can therefore be conducted purposefully to save technical and human resources.

IL-6 might serve as a decision support to assess the severity of pulmonary involvement in patients with respiratory infections. Especially in an ambulant setting without chest CT availability, IL-6 as a simply and quickly determinable marker of routine laboratory diagnostics may be used as a decision tool to help estimate the extent of the disease. This information allows further diagnostic and therapeutic steps to be swiftly and efficiently performed. Additionally, in times of restricted capabilities in our health system, the number of performed chest CT scans might be reduced.

Due to the rather small sample size, the conclusions cannot claim general validity, but are rather hypothesis-forming, paving the way for future research. Even though the emergency room setting was chosen to ensure a broad range of patients, the design as a single-center study may limit the generalizability of the results, not fully accounting for regional, demographic, and ethnic differences. While a healthy control group was included to enable comparisons to healthy people, its suitability may be limited due to its collection at the emergency room. In synopsis, the applicability of the study’s conclusions to broader populations or to different settings of patient care may suffer, limiting the study’s representativity.

Further studies with more participants will be needed to conduct external validation of the findings by reproducing the research in an independent cohort. Presumably, a multi-centre study may be conducted to include diversity in the study population and provide more resilient and generalizable data in the future*.* In addition, future research should consider potential biomarker changes over time and progression of COVID-19 and other respiratory infections by collecting longitudinal data. In addition, the potential of DPP3 as a COVID-19 specific biomarker could be validated in conjunction with other established COVID-19 biomarkers in the future.

## Conclusion

DPP3 might be able to detect a COVID-19 infection in an ambulant setting promptly. IL-6 might help estimate the degree of pulmonary involvement in respiratory patients in case CT imaging is unavailable.

## Limitations

The number of recruited patients with COVID-19 (n = 114) and without COVID-19 (n = 35) is relatively low and may affect the generalizability of the results.

The single-centre study may not fully account for regional, demographic, and ethnic differences. Since the study population may not be fully representative of all COVID-19 cases and other respiratory infections, a selection bias cannot be completely excluded.

As the patients in the healthy control group presented themselves in the emergency room and showed significant differences to the respiratory patients regarding the baseline data, they may not fully meet the criteria of a perfectly healthy and comparable control group.

Since patients were included between March 2020 and June 2021, SARS-CoV-2 variants in the study were presumably different from the predominant variants at the time of publication. Even though relevant viral features were consistent over time and between variants, studies including COVID-19 patients with the latest SARS-CoV-2 variants may be needed to confirm the current results.

## Data Availability

Please contact author for data requests.

## References

[1] (2023) China: WHO Coronavirus Disease (COVID-19) Dashboard With Vaccination Data. https://covid19.who.int/region/wpro/country/cn. Accessed 20 Jan 2023

[2] Lee CM, Snyder SH (1982). Dipeptidyl-aminopeptidase III of rat brain. Selective affinity for enkephalin and angiotensin. J Biol Chem.

[3] Prajapati SC, Chauhan SS (2011). Dipeptidyl peptidase III: a multifaceted oligopeptide N-end cutter. FEBS J.

[4] Rehfeld L, Funk E, Jha S, Macheroux P, Melander O, Bergmann A (2019). Novel methods for the quantification of dipeptidyl peptidase 3 (DPP3) concentration and activity in human blood samples. J Appl Lab Med.

[5] Blet A, Deniau B, Santos K (2021). Monitoring circulating dipeptidyl peptidase 3 (DPP3) predicts improvement of organ failure and survival in sepsis: a prospective observational multinational study. Crit Care.

[6] Dépret F, Amzallag J, Pollina A (2020). Circulating dipeptidyl peptidase-3 at admission is associated with circulatory failure, acute kidney injury and death in severely ill burn patients. Crit Care.

[7] Frigyesi A, Lengquist M, Spångfors M (2021). Circulating dipeptidyl peptidase 3 on intensive care unit admission is a predictor of organ dysfunction and mortality. J Intensive Care.

[8] Tipnis SR, Hooper NM, Hyde R, Karran E, Christie G, Turner AJ (2000). A human homolog of angiotensin-converting enzyme. Cloning and functional expression as a captopril-insensitive carboxypeptidase. J Biol Chem.

[9] Moon J-Y (2013). Recent update of renin-angiotensin-aldosterone system in the pathogenesis of hypertension. Electrolyte Blood Press.

[10] Li W, Moore MJ, Vasilieva N (2003). Angiotensin-converting enzyme 2 is a functional receptor for the SARS coronavirus. Nature.

[11] Hoffmann M, Kleine-Weber H, Schroeder S (2020). SARS-CoV-2 cell entry depends on ACE2 and TMPRSS2 and is blocked by a clinically proven protease inhibitor. Cell.

[12] van Lier D, Deniau B, Santos K (2023). Circulating dipeptidyl peptidase 3 and bio-adrenomedullin levels are associated with impaired outcomes in critically ill COVID-19 patients: a prospective international multicentre study. ERJ Open Res.

[13] Heinicke U, Adam E, Sonntagbauer M, von Knethen A, Zacharowski K, Neb H (2020). Angiotensin II treatment in COVID-19 patients: More risk than benefit? A single-center experience. Crit Care.

[14] Calandra T, Gerain J, Heumann D, Baumgartner JD, Glauser MP (1991). High circulating levels of interleukin-6 in patients with septic shock: evolution during sepsis, prognostic value, and interplay with other cytokines. The Swiss-Dutch J5 Immunoglobulin Study Group. Am J Med.

[15] Bacci MR, Leme RCP, Zing NPC (2015). IL-6 and TNF-α serum levels are associated with early death in community-acquired pneumonia patients. Braz J Med Biol Res.

[16] Glynn P, Coakley R, Kilgallen I, Murphy N, O'Neill S (1999). Circulating interleukin 6 and interleukin 10 in community acquired pneumonia. Thorax.

[17] Martínez R, Menéndez R, Reyes S (2011). Factors associated with inflammatory cytokine patterns in community-acquired pneumonia. Eur Respir J.

[18] Han H, Ma Q, Li C (2020). Profiling serum cytokines in COVID-19 patients reveals IL-6 and IL-10 are disease severity predictors. Emerg Microbes Infect.

[19] Potere N, Batticciotto A, Vecchié A (2021). The role of IL-6 and IL-6 blockade in COVID-19. Expert Rev Clin Immunol.

[20] Chen L-D, Zhang Z-Y, Wei X-J (2020). Association between cytokine profiles and lung injury in COVID-19 pneumonia. Respir Res.

[21] Homayounieh F, Bezerra Cavalcanti Rockenbach MA, Ebrahimian S (2021). Multicenter assessment of CT pneumonia analysis prototype for predicting disease severity and patient outcome. J Digit Imaging.

[22] Gouda W, Yasin R (2020). COVID-19 disease: CT pneumonia analysis prototype by using artificial intelligence, predicting the disease severity. Egypt J Radiol Nucl Med.

[23] Huang L, Han R, Ai T (2020). Serial quantitative chest CT assessment of COVID-19: a deep learning approach. Radiol Cardiothorac Imaging.

[24] Ardali Duzgun S, Durhan G, Basaran Demirkazik F (2021). AI-based quantitative CT analysis of temporal changes according to disease severity in COVID-19 pneumonia. J Comput Assist Tomogr.

[25] Okuma T, Hamamoto S, Maebayashi T (2021). Quantitative evaluation of COVID-19 pneumonia severity by CT pneumonia analysis algorithm using deep learning technology and blood test results. Jpn J Radiol.

[26] Karami H, Derakhshani A, Ghasemigol M (2021). Weighted gene co-expression network analysis combined with machine learning validation to identify key modules and hub genes associated with SARS-CoV-2 infection. J Clin Med.

[27] Schlossbauer MH, Hubauer U, Stadler S (2019). The role of the tubular biomarkers NAG, kidney injury molecule-1 and neutrophil gelatinase-associated lipocalin in patients with chest pain before contrast media exposition. Biomark Med.

[28] Gustine JN, Jones D (2021). Immunopathology of Hyperinflammation in COVID-19. Am J Pathol.

[29] Magliocca A, Omland T, Latini R (2020). Dipeptidyl peptidase 3, a biomarker in cardiogenic shock and hopefully much more. Eur J Heart Fail.

[30] Takagi K, Blet A, Levy B (2020). Circulating dipeptidyl peptidase 3 and alteration in haemodynamics in cardiogenic shock: results from the OptimaCC trial. Eur J Heart Fail.

[31] Deniau B, Blet A, Santos K (2020). Inhibition of circulating dipeptidyl-peptidase 3 restores cardiac function in a sepsis-induced model in rats: a proof of concept study. PLoS ONE.

[32] Deniau B, Rehfeld L, Santos K (2020). Circulating dipeptidyl peptidase 3 is a myocardial depressant factor: dipeptidyl peptidase 3 inhibition rapidly and sustainably improves haemodynamics. Eur J Heart Fail.

[33] Miesbach W (2020). Pathological role of angiotensin II in severe COVID-19. TH Open.

[34] Ekholm M, Kahan T, Jörneskog G, Bröijersen A, Wallén NH (2009). Angiotensin II infusion in man is proinflammatory but has no short-term effects on thrombin generation in vivo. Thromb Res.

[35] Kishimoto T (1989). The biology of interleukin-6. Blood.

[36] Kishimoto T, Akira S, Taga T (1992). Interleukin-6 and its receptor: a paradigm for cytokines. Science.

[37] Akira S, Taga T, Kishimoto T (1993). Interleukin-6 in biology and medicine. Adv Immunol.

[38] Gauldie J, Richards C, Harnish D, Lansdorp P, Baumann H (1987). Interferon beta 2/B-cell stimulatory factor type 2 shares identity with monocyte-derived hepatocyte-stimulating factor and regulates the major acute phase protein response in liver cells. Proc Natl Acad Sci U S A.

[39] Kishimoto T, Akira S, Narazaki M, Taga T (1995). Interleukin-6 family of cytokines and gp130. Blood.

[40] Jones SA (2005). Directing transition from innate to acquired immunity: defining a role for IL-6. J Immunol.

[41] Damas P, Ledoux D, Nys M (1992). Cytokine serum level during severe sepsis in human IL-6 as a marker of severity. Ann Surg.

[42] Andrijevic I, Matijasevic J, Andrijevic L, Kovacevic T, Zaric B (2014). Interleukin-6 and procalcitonin as biomarkers in mortality prediction of hospitalized patients with community acquired pneumonia. Ann Thorac Med.

[43] Zobel K, Martus P, Pletz MW (2012). Interleukin 6, lipopolysaccharide-binding protein and interleukin 10 in the prediction of risk and etiologic patterns in patients with community-acquired pneumonia: results from the German competence network CAPNETZ. BMC Pulm Med.

[44] Ramírez P, Ferrer M, Martí V (2011). Inflammatory biomarkers and prediction for intensive care unit admission in severe community-acquired pneumonia. Crit Care Med.

[45] Meduri GU, Headley S, Kohler G (1995). Persistent elevation of inflammatory cytokines predicts a poor outcome in ARDS. Plasma IL-1 beta and IL-6 levels are consistent and efficient predictors of outcome over time. Chest.

[46] Bauer TT, Montón C, Torres A (2000). Comparison of systemic cytokine levels in patients with acute respiratory distress syndrome, severe pneumonia, and controls. Thorax.

[47] Nishimoto N, Yoshizaki K, Miyasaka N (2004). Treatment of rheumatoid arthritis with humanized anti-interleukin-6 receptor antibody: a multicenter, double-blind, placebo-controlled trial. Arthritis Rheum.

[48] Yokota S, Imagawa T, Mori M (2008). Efficacy and safety of tocilizumab in patients with systemic-onset juvenile idiopathic arthritis: a randomised, double-blind, placebo-controlled, withdrawal phase III trial. Lancet.

[49] Villaescusa L, Zaragozá F, Gayo-Abeleira I, Zaragozá C (2022). A new approach to the management of COVID-19. Antagonists of IL-6: siltuximab. Adv Ther.

[50] Du P, Geng J, Wang F, Chen X, Huang Z, Wang Y (2021). Role of IL-6 inhibitor in treatment of COVID-19-related cytokine release syndrome. Int J Med Sci.

[51] Ye Z, Zhang Y, Wang Y, Huang Z, Song B (2020). Chest CT manifestations of new coronavirus disease 2019 (COVID-19): a pictorial review. Eur Radiol.

[52] Pan Y, Guan H, Zhou S (2020). Initial CT findings and temporal changes in patients with the novel coronavirus pneumonia (2019-nCoV): a study of 63 patients in Wuhan, China. Eur Radiol.

[53] Song F, Shi N, Shan F (2020). Emerging 2019 novel coronavirus (2019-nCoV) pneumonia. Radiology.

[54] Bernheim A, Mei X, Huang M (2020). Chest CT findings in coronavirus disease-19 (COVID-19): relationship to duration of infection. Radiology.

[55] Wu J, Wu X, Zeng W (2020). Chest CT findings in patients with coronavirus disease 2019 and its relationship with clinical features. Invest Radiol.

[56] Shi H, Han X, Jiang N (2020). Radiological findings from 81 patients with COVID-19 pneumonia in Wuhan, China: a descriptive study. Lancet Infect Dis.

[57] Li K, Wu J, Wu F (2020). The clinical and chest CT features associated with severe and critical COVID-19 pneumonia. Investig Radiol.

